# Effect of Rosmarinic Acid and Sinapic Acid on Oxidative Stress Parameters in the Cardiac Tissue and Serum of Type 2 Diabetic Female Rats

**DOI:** 10.3390/antiox8120579

**Published:** 2019-11-22

**Authors:** Maria Zych, Weronika Wojnar, Sławomir Borymski, Katarzyna Szałabska, Piotr Bramora, Ilona Kaczmarczyk-Sedlak

**Affiliations:** 1 Department of Pharmacognosy and Phytochemistry, Faculty of Pharmaceutical Sciences in Sosnowiec, Medical University of Silesia, Katowice, Jagiellońska 4, 41-200 Sosnowiec, Poland; wwojnar@sum.edu.pl (W.W.); katarzyna.szalabska@gmail.com (K.S.); piotr.bramora@med.sum.edu.pl (P.B.); isedlak@sum.edu.pl (I.K.-S.); 2 Faculty of Natural Sciences, Institute of Biology, Biotechnology and Environmental Protection, University of Silesia, Jagiellońska 28, 40-032 Katowice, Poland; slawomir.borymski@us.edu.pl

**Keywords:** type 2 diabetes, female rats, oxidative stress, cardiac tissue, rosmarinic acid, sinapic acid, glucose homeostasis, lipid profile

## Abstract

Cardiovascular diseases are one of the most common complications of type 2 diabetes. They are considered the leading cause of death among diabetics. One of the mechanisms underlying diabetic cardiovascular complications is oxidative stress. Many phenolic acids are regarded as antioxidants. The aim of the study was to investigate the effect of rosmarinic acid (RA) and sinapic acid (SA) on oxidative stress parameters in the cardiac tissue and serum of type 2 diabetic female rats. Additionally, the effect of these compounds on glucose homeostasis and lipid profile in the serum was evaluated. Type 2 diabetes was induced with high-fat diet and streptozotocin. RA at the doses of 10 and 50 mg/kg and SA at the doses of 5 and 25 mg/kg were administrated orally for 28 days. Untreated diabetic rats exhibited unfavorable changes in glucose metabolism and lipid profile. Changes in the enzymatic and non-enzymatic markers indicated the onset of oxidative stress in these animals. The results showed that the higher doses of the tested phenolic acids—50 mg/kg of RA and 25 mg/kg of SA—revealed beneficial effects on oxidative stress in the cardiac tissue of diabetic rats.

## 1. Introduction

Diabetes mellitus (DM) poses a serious public health issue [1]. As of 2017, there were 425 million diagnosed diabetics worldwide, and this number may increase up to 629 million in 2045 according to an International Diabetes Federation report [2]. Typically, diabetes can be classified as type 1 diabetes, type 2 diabetes, gestational diabetes, or as other types with different etiologies. Type 2 diabetes, which is characterized by hyperglycemia, insulin resistance, and disturbance in lipid, protein, and carbohydrate metabolism, is the most often diagnosed type in adults (about 90% of adult diabetics) [3,4,5,6]. In patients with diabetes, a range of diabetic complications may develop, namely diabetic cardiomyopathy, myocardial insufficiency, retinopathy, nephropathy, neuropathy, and hyperlipidemia [5,7,8]. However, the cardiovascular complications of diabetes are considered the major factor resulting in deaths of diabetic patients [3,7,9,10].

It is believed that diabetic complications, such as diabetic-related cardiac dysfunctions, are directly connected with redox imbalance and oxidative stress [9,11,12,13,14]. Oxidative stress can be described as the lack of ability of the organism to neutralize free radicals, accompanied by overproduction of the latter. When the endogenous antioxidative system becomes insufficient in order to cope with the excess of free radicals, it could be helpful to aid antioxidative capabilities of the body with exogenous antioxidants [15]. Numerous scientific reports indicate that exogenous antioxidants improved oxidative stress-related parameters in the serum and various tissues [16,17,18,19,20,21,22], including cardiac tissue [23,24,25,26,27], in animals with experimentally-induced diabetes. Phenolic acids—rosmarinic acid and sinapic acid—may be considered as such exogenous antioxidants.

Both rosmarinic acid and sinapic acid are compounds commonly occurring in plants. These phenolic acids can be delivered to the organism on a daily basis in numerous herbs, vegetables, and fruits. Rosmarinic acid can be found mainly in Lamiaceae plants, such as rosemary, peppermint, lemon balm, or sage [28,29], whereas sinapic acid occurs in citrus fruits, strawberries, and Brassicaceae vegetables such as broccoli, turnip, kale, or tronchuda cabbage [30,31].

Scientific literature shows that both rosmarinic and sinapic acids have a beneficial effect on parameters connected with the glucose metabolism in diabetic rats [32,33,34,35,36,37,38]. Additionally, rosmarinic acid positively affects several oxidative stress-related parameters and lipid profile in rats with experimentally induced diabetes [32,33]. Both these phenolic acids reveal a favorable effect on lipid metabolism and parameters related to oxidative stress in the serum of rats in the early stage of estrogen deficiency [39,40]. What is more, there are reports indicating a beneficial effect of rosmarinic acid and sinapic acid on cardiac dysfunction in several experimental rodent models [41,42,43,44,45].

Based on scientific reports, we hypothesize that disturbance in redox balance in the serum and cardiac tissue may develop in long-term hyperglycemia in type 2 diabetic rats. Moreover, we suppose that rosmarinic acid and sinapic acid could mitigate hyperglycemia and hyperlipidemia and they may act as antioxidants in cardiac tissue. Thus, this experiment was conducted in order to evaluate the effect of rosmarinic acid and sinapic acid on the parameters connected with glucose and lipid metabolism in the serum and on the markers of oxidative stress in the serum and cardiac tissue of female rats with type 2 diabetes induced with high-fat diet and streptozotocin.

## 2. Materials and Methods 

### 2.1. Animals and Drugs

The experiment was carried out on sexually mature (3-month-old) female Wistar rats. The study was approved by the Local Ethics Commission in Katowice, Poland (permission numbers: 38/2015, 148/2015 and 66/2016). The rats were provided by Center of Experimental Medicine, Medical University of Silesia (Katowice, Poland). The animals were kept in standard laboratory conditions (EU Directive 2010/63).

The following drugs and substances were used in the experiment: streptozotocin (STZ, Cayman Chemical Company, Ann Arbor, MI, USA); ketamine (Ketamina 10%, Biowet Puławy Sp. z o. o., Puławy, Poland); xylazine (Xylapan, Vetoquinol Biowet, Gorzów Wlkp., Poland); rosmarinic acid (Sigma-Aldrich, St. Louis, MO, USA); and sinapic acid (Sigma-Aldrich, St. Louis, MO, USA).

During a one-week acclimation period the animals were randomly assigned to six experimental groups (*n* = 10):

- Control, non-diabetic rats (C group)
- High-fat diet and streptozotocin (HFD + STZ)-induced type 2 diabetic control rats (DM2 group)
- HFD + STZ diabetic rats administered orally with rosmarinic acid at a dose of 10 mg/kg (RA10 group)
- HFD + STZ diabetic rats administered orally with rosmarinic acid at a dose of 50 mg/kg (RA50 group)
- HFD + STZ diabetic rats administered orally with sinapic acid at a dose of 5 mg/kg (SA5 group)
- HFD + STZ diabetic rats administered orally with sinapic acid at a dose of 25 mg/kg (SA25 group)

**Figure 1:**
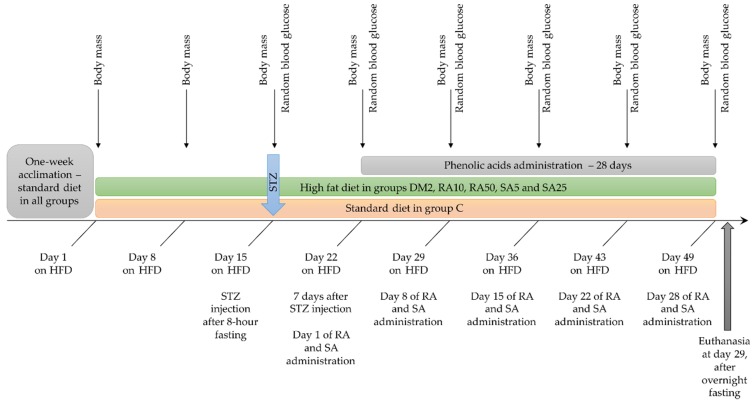
Timeline of the in vivo part of the experiment. HFD: high-fat diet; STZ: streptozotocin; RA: rosmarinic acid; SA: sinapic acid; C: control rats; DM2: HFD + STZ-induced type 2 diabetic rats; RA10 and RA50: HFD + STZ-induced diabetic rats treated with rosmarinic acid at a dose of 10 mg/kg and 50 mg/kg, respectively; SA5 and SA25: HFD + STZ-induced diabetic rats treated with sinapic acid at a dose of 5 mg/kg and 25 mg/kg, respectively, for 28 days.

The animals were weighted at the beginning of the experiment, and then once a week. The last weight record was taken before an overnight fasting. From the day of STZ injection, a random blood glucose level from tail capillary vessels (obtained by cutting the end of the tail) was measured once a week by MicroDot glucometer equipped with test strips (Cambridge Sensor USA, Plainfield, IL, USA). During the whole experiment the animals had unlimited water supply and appropriate chow: rats in the C group were fed with a standard laboratory chow (Labofeed B, Wytwórnia Pasz “Morawski”, Kcynia, Poland) and the rats in the DM2, RA10, RA50, SA5, and SA25 groups were fed for 2 weeks before STZ injection with high-fat diet (HFD) enriched with 32% of fat from lard (Wytwórnia Pasz “Morawski”, Kcynia, Poland). The HFD in these groups was continued till the end of the experiment. The overall duration of HFD in the groups with experimentally induced type 2 diabetes was 49 days. Before a single injection with streptozotocin (40 mg/kg intraperitoneally) the rats were fasted for 8 h. Streptozotocin was dissolved in 0.1 M citric buffer and 1 mL/kg of this solution was injected. The rats from the C group were injected with an appropriate volume of 0.1 M citric buffer only. Seven days after the STZ injection random blood glucose level was measured. If the result of this test was higher than 200 mg/dL, the animal was considered diabetic. Administration of rosmarinic acid and sinapic acid started 7 days after STZ injection and lasted for 28 days. The phenolic acids suspended in water (with addition of Tween 20 quantum satis, max. 1 µL of Tween 20 per 1 mL of water) were administered by gavage once a day. Control rats from the C and DM2 groups received water with Tween 20 by gavage at the same volume as the rats from the rosmarinic acid and sinapic acid-treated groups received the suspension of phenolic acids (2 mL/kg). The timeline of the in vivo part of the experiment is presented in the Figure 1.

24 h after the administration of the last dose of phenolic acids and overnight fasting, rats were euthanized by injection of ketamine and xylazine mixture and cardiac exsanguination. In order to separate the serum required for biochemical assays, the blood obtained from the heart was centrifuged. Vital organs such as the heart, liver, right kidney and the uterus were excised from the animals and weighted. Then, each heart was homogenized separately in phosphate buffered saline (PBS), pH 7.4 (10% w/v), and centrifuged (10,000× *g*, 15 min, +4 °C). The obtained supernatant was used for further examinations. All assays were measured in Tecan Infinite M200 PRO plate reader with Magellan 7.2 software (Tecan Austria, Grödig, Austria).

### 2.2. Biochemical Assays in the Serum

The levels of the estradiol and interleukin 18 (IL-18) were assessed with ELISA kits (DiaMetra, Segrate-Milano, Italy for estradiol and Cloud-Clone Corp. Houston, TX, USA for IL-18). Aspartate aminotransferase (AST) and alanine aminotransferase (ALT) activities as well as uric acid and urea levels were measured spectrophotometrically with Biosystems kits (Costa Brava, Barcelona, Spain); the creatinine was assayed with Pointe Scientific Inc. kit (Canton, MI, USA).

### 2.3. Parameters Connected with Glucose Homeostasis in the Serum and Cardiac Tissue

Glucose and fructosamine levels in the serum were evaluated with Pointe Scientific Inc. kits. Insulin levels in the serum were measured with the BioVendor ELISA kit (Brno, Czech Republic). The homeostasis model assessment of insulin resistance (HOMA-IR) was calculated according to the following Equation:

HOMA-IR = (fasting glucose (mg/dL) × fasting insulin (μU/mL)/405),

while the quantitative insulin sensitivity check index (QUICKI) was calculated as follows:

QUICKI = 1/(log(fasting insulin μU/mL) + log(fasting glucose mg/dL))

Advanced glycation end products (AGEs) in both the serum and cardiac tissue were assayed with ELISA method (Cell Biolabs, San Diego, CA, USA).

### 2.4. Lipid Profile Assessment in the Serum

Total cholesterol, triglycerides, low-density lipoprotein cholesterol (LDL-C), and high-density lipoprotein cholesterol (HDL-C) levels were assayed spectrophotometrically with Pointe Scientific Inc kits (Canton, MI, USA).

### 2.5. Oxidative Stress-Related Parameters Assessment in the Serum and Cardiac Tissue

Activity of superoxide dismutase (SOD) and catalase (CAT) was measured in both the serum and cardiac tissue. Additionally, glutathione peroxidase (GPx) activity was evaluated in cardiac tissue. All the enzymes were assayed with the use of Cayman kits (Cayman Chemical, Ann Arbor, MI, USA) and their activity was expressed per 1 mg of protein. The protein level was assessed with the Pointe Scientific Inc kit (Canton, MI, USA).

Non-protein sulfhydryl groups (NPSH) levels in the serum and cardiac tissue were examined according to the protocol proposed by Sedlak and Lindsay [46]. In this method, a colorful product of reaction between sulfhydryl groups and 5,5’-dithiobis-(2-nitrobenzoic acid) (Sigma-Aldrich, St Louis, MO, USA) is measured at 412 nm. The standard curve was prepared with reduced glutathione (Sigma-Aldrich, St Louis, MO, USA). 

Thiobarbituric acid reactive substances (TBARS) levels in the serum and cardiac tissue were evaluated with the Buege and Aust [47] method, in which thiobarbituric acid reacts with lipid peroxidation products, and the colorful product of this reaction is measured spectrophotometrically at 535 nm. Obtained results were calculated with the use of the extinction coefficient = 1.56 × 10^5^/M cm.

The level of advanced oxidation protein products (AOPP) was obtained according to the Witko–Sarsat method [48], and chloramine T (Sigma-Aldrich, St Louis, MO, USA) was used for standard curve. The absorbance was read at 340 nm. In the serum, the AOPP level was expressed as 1 nmol of chloramine T equivalent per 1 mL and in the cardiac tissue as 1 nmol of chloramine T equivalent per 1 mg of protein.

### 2.6. Statistical Analyses 

The results are presented as arithmetical means ± SEM. To evaluate statistical significance, one-way ANOVA followed by Fisher’s Least Significant Difference (LSD) post-hoc test was used (Statistica 13 software, StatSoft, Kraków, Poland). The results were considered significant if *p* < 0.05. Additionally, data obtained from the cardiac tissue and serum were subjected to principal component analysis (PCA) using PAST 3.26 software [49]. The PCA data was subjected to MANOVA (Statistica 13; Appendix A).

The number of rat subjects in each experimental group was equal at the beginning of the experiment (*n* = 10). However, in the DM2 control and phenolic acids-treated groups the number of rats varied based on the diabetes onset—only the rats classified as type 2 diabetic were included in analyses. After the classification, the final numbers were as follows: DM2, *n* = 7; RA10, *n* = 8; RA50, *n* = 6; SA5, *n* = 7; and SA25, *n* = 7, whereas C remained at *n* = 10. 

## 3. Results

### 3.1. Effect of Rosmarinic Acid and Sinapic Acid on Body Mass and Mass of the Vital Organs

**Figure 2:**
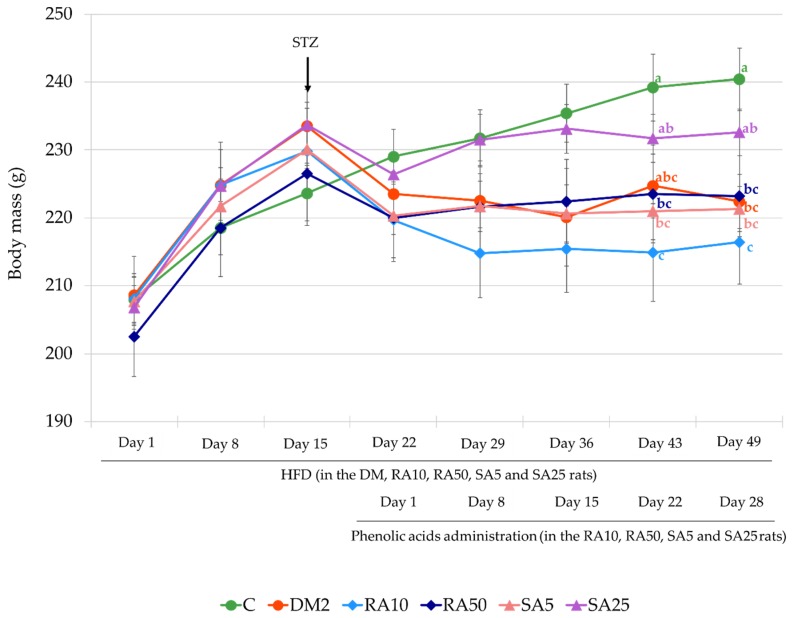
Effect of rosmarinic acid and sinapic acid on the body mass of the HFD + STZ-induced type 2 diabetic rats. The results presented as mean ± SEM were evaluated by one-way ANOVA followed by the LSD post-hoc test (statistically significant if *p* < 0.05). Values which have the same superscript letter(s) in each time point are not significantly different. When no letters are present in the superscript the ANOVA test was not significant. C: control rats; DM2: HFD + STZ-induced diabetic rats; RA10 and RA50: HFD + STZ-induced diabetic rats treated with rosmarinic acid at a dose of 10 mg/kg and 50 mg/kg, respectively; SA5 and SA25: HFD + STZ-induced diabetic rats treated with sinapic acid at a dose of 5 mg/kg and 25 mg/kg, respectively, for 28 days; HFD: high-fat diet; STZ: streptozotocin.

At the beginning of the experiment, before HFD introduction, the mean body mass of the animals in all the experimental groups was comparable. There was no difference in the mean body mass between the rats in all groups after being fed with HFD for 7 and 14 days. On the first day of phenolic acid administration (7 days after STZ injection) as well as after 8 and 15 days of rosmarinic and sinapic acid treatment there were no differences in the mean body mass of all the rats. On day 22 of phenolic acids treatment it was noted that the mean body mass of the DM2 rats was not significantly different from the mean body mass of the C rats, but the mean body mass of the rats from the RA10, RA50, and SA5 groups was significantly lower than in the rats in the C group. On day 28 (the last day of the treatment with rosmarinic acid and sinapic acid) a significant decrease in the mean body mass of the rats from the DM2, RA10, RA50, and SA5 groups in comparison with the C group rats was observed. The mean body mass of the SA25 rats was not significantly different from the mean body mass of the rats from the C and DM2 groups (Figure 2).

In the DM2 rats the mean mass of the heart and liver was significantly higher than in the rats from the C group. The mass of the remaining organs did not differ between these groups. Administration of both rosmarinic acid and sinapic acid for 28 days did not alter the mass of any vital organ tested, as compared to the DM2 group of rats (Appendix B, Table A1).

### 3.2. Effect of Rosmarinic Acid and Sinapic Acid on Biochemical Parameters in the Serum

In the serum of the rats with HFD + STZ-induced type 2 diabetes, a decrease in the level of interleukin 18 (IL-18) and urea, as well as an increase in the ALT activity was observed when compared to the rats from the C group. No significant differences were noted in the level of estradiol, uric acid, and creatinine or the activity of the AST after diabetes induction in comparison with the non-diabetic control rats. Administration of phenolic acids at both doses did not affect any of the tested parameters when compared to the rats from the DM2 group. The IL-18 level and the ALT activity in the serum remained significantly lower in all the phenolic acid-treated groups than in the serum of the C rats. Only in the RA50 and SA5 groups of rats there were no significant differences in the urea levels in the serum as compared to the C rats (Appendix B, Table A2).

### 3.3. Effect of Rosmarinic Acid and Sinapic Acid on Parameters Connected with the Glucose Homeostasis in the Serum and Cardiac Tissue

**Figure 3:**
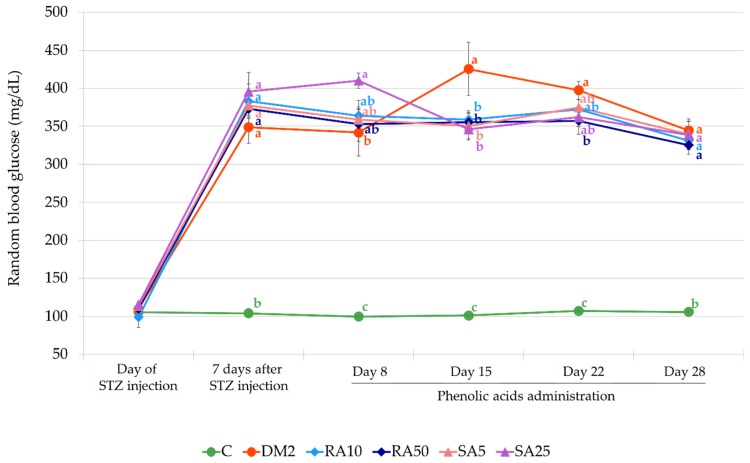
Effect of rosmarinic acid and sinapic acid on the random blood glucose level of the HFD + STZ-induced type 2 diabetic rats. The results presented as mean ± SEM were evaluated by one-way ANOVA followed by LSD post-hoc test (statistically significant if *p* < 0.05). Values which have the same superscript letter(s) in each time point are not significantly different. When no letters are present in the superscript, the ANOVA test was not significant. C: control rats; DM2: HFD + STZ-induced diabetic rats; RA10 and RA50: HFD + STZ-induced diabetic rats treated with rosmarinic acid at a dose of 10 mg/kg and 50 mg/kg, respectively; SA5 and SA25: HFD + STZ-induced diabetic rats treated with sinapic acid at a dose of 5 mg/kg and 25 mg/kg, respectively, for 28 days; HFD: high-fat diet; STZ: streptozotocin.

At the day of STZ injection (14 days after HFD introduction) the mean random blood glucose level in all groups of rats was comparable. From the first day of phenolic acids administration (i.e., 7 days after STZ injection) until the end of the experiment, the random blood glucose level in all the groups of rats which were fed with HFD and injected with STZ (DM2, RA10, RA50, SA5, and SA25) was significantly higher than in the non-diabetic control rats. On the day 15 of treatment with both the doses of rosmarinic acid and sinapic acid a significant decrease of the random blood glucose level was observed when compared to the DM2 rats, and on day 22, only the higher dose of rosmarinic acid (50 mg/kg) significantly reduced the random blood glucose level in comparison with the DM2 rats. At the end of the experiment, before an overnight fasting, the random glucose level in all the phenolic acid-treated groups was not different from the random blood glucose level in the DM2 rats (Figure 3).

The fasting glucose and fructosamine levels in the serum as well as the HOMA-IR were significantly higher and the QUICKI was lower in the DM2 rats than in the C rats. The levels of insulin and AGEs in the serum of the DM2 rats as well as AGEs level in the cardiac tissue from the animals of the same experimental group were not statistically different from their levels observed in the C rats. Administration of rosmarinic acid at the dose of 50 mg/kg and sinapic acid at the dose of 25 mg/kg for 28 days resulted in a significant decrease of the fasting glucose level in comparison with the DM2 rats, however this parameter remained significantly higher than in the rats form the C group. Treatment with the lower doses of phenolic acids did not affect fasting glucose level. The remainder of the parameters connected with glucose homeostasis in the serum or the cardiac tissue remained unchanged after phenolic acids administration to the diabetic rats (Table 1).

### 3.4. Effect of Rosmarinic Acid and Sinapic Acid on the Lipid Profile in the Serum 

**Figure 4:**
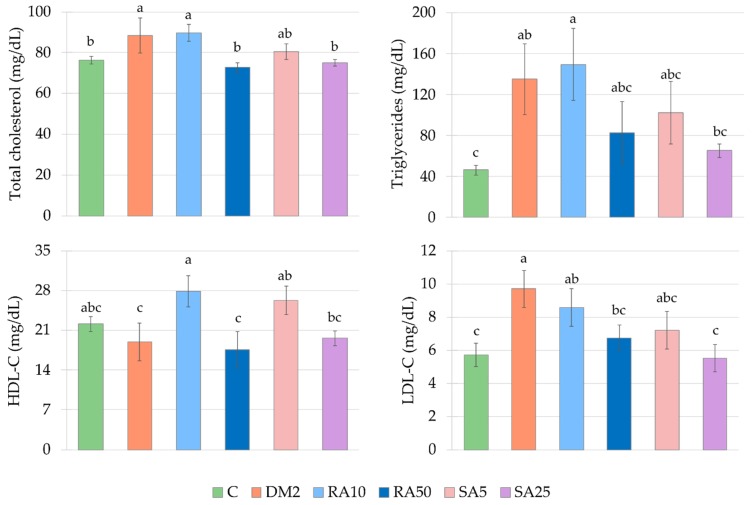
Effect of rosmarinic acid and sinapic acid on the lipid profile in the serum of the HFD + STZ-induced type 2 diabetic rats. The results presented as mean ± SEM were evaluated by one-way ANOVA followed by LSD post-hoc test (statistically significant if *p* < 0.05). Values which have the same superscript letter(s) in each graph are not significantly different. When no letters are present in the superscript, the ANOVA test was not significant. C: control rats; DM2: HFD + STZ-induced diabetic rats; RA10 and RA50: HFD + STZ-induced diabetic rats treated with rosmarinic acid at a dose of 10 mg/kg and 50 mg/kg, respectively; SA5 and SA25: HFD + STZ-induced diabetic rats treated with sinapic acid at a dose of 5 mg/kg and 25 mg/kg, respectively, for 28 days; HFD: high-fat diet; STZ: streptozotocin; HDL-C: high-density lipoprotein cholesterol; LDL-C: low-density lipoprotein cholesterol.

There were significantly higher total cholesterol, triglycerides and LDL-cholesterol levels in the serum of the control type 2 diabetic rats than in the control healthy rats. The HDL-cholesterol level was not changed in the DM2 rats as compared with the C rats. Administration of the lower doses of tested phenolic acids, i.e., 10 mg/kg of rosmarinic acid and 5 mg/kg of sinapic acid, resulted in an increase in the HDL-C level, while administration of higher doses (50 mg/kg of rosmarinic acid and 25 mg/kg of sinapic acid) effected in a decrease of the total cholesterol and LDL-C levels, when compared to the DM2 rats. There was no significant change in the triglycerides level between all the phenolic acid-treated groups of rats and DM2 rats. In the RA10 group of rats the levels of total cholesterol, triglycerides, and LDL-C were significantly higher than in the C rats, and as far as other doses of phenolic acids and other lipid profile parameters are concerned, there were no differences in comparison with the C rats (Figure 4).

### 3.5. Effect of Rosmarinic Acid and Sinapic Acid on Oxidative Stress Parameters in the Serum

Induction of diabetes by HFD and STZ resulted in a disturbance in oxidative stress parameters in the serum of the rats as compared to the rats from the non-diabetic group C. In the DM2 rats there was a significant increase in the SOD and CAT activities as well as in the TBARS level. The diabetes induction did not affect the NPSH or AOPP levels in the serum, when compared to the C rats. Administration of phenolic acids at both the doses to the diabetic rats did not change any of the examined oxidative stress-related parameters in the serum. Moreover, in the RA10 rats there were no differences in the SOD and CAT activities in comparison with the C rats, and in the SA25 rats no differences in the TBARS level in relation to the C rats were noted (Table 2).

### 3.6. PCA for the Serum

**Figure 5:**
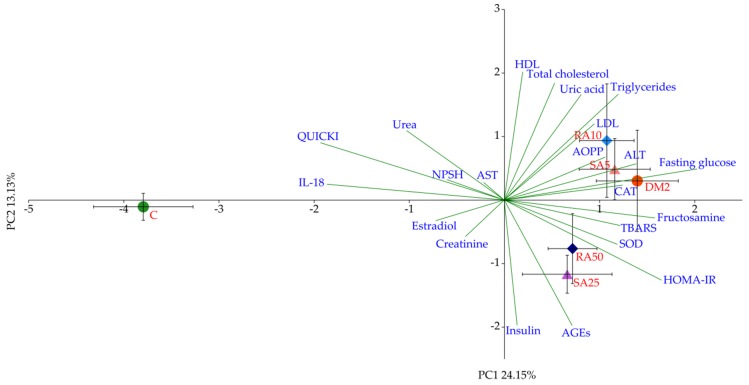
Principal component analysis (PCA) biplot of parameters obtained from the serum. Symbols and red fonts indicate means for individual experimental groups (whiskers represent SEM). ●—Control groups: green—untreated control rats (C); red—HFD + STZ control rats (DM2). ◆—Rosmarinic acid-treated HFD + STZ rats: light blue—10 mg/kg (RA10), navy blue – 50 mg/kg (RA50). ▲—Sinapic acid-treated HFD + STZ rats: pink—5 mg/kg (SA5), purple—25 mg/kg (SA25). Blue fonts and green lines show correlations of measured parameters with regard to experimental groups in the PCA plot (autoscaled). AST: aspartate aminotransferase; IL-18: interleukin-18.

The PCA from the serum showed a prominent separation of groups across PC1, accounting for more than 24% of observed variation. The C group separated sharply to the left, whereas the remaining groups, DM2 and rats treated with phenolic acids, clustered on the right-hand side of the plot. This separation was statistically significant (*p* < 0.001, LSD), whereas minor clustering between other groups was not, with regard to both the PC1 and PC2 axes. The main variables contributing to the separation across PC1 were fasting glucose and fructosamine concentrations followed by HOMA-IR, correlating with the DM2 group on the right-hand side, whereas the IL-18 level and the QUICKI correlated with the C group, on the left-hand side of the plot (Figure 5).

### 3.7. Effect of Rosmarinic Acid and Sinapic Acid on Oxidative Stress Parameters in the Cardiac Tissue

**Figure 6:**
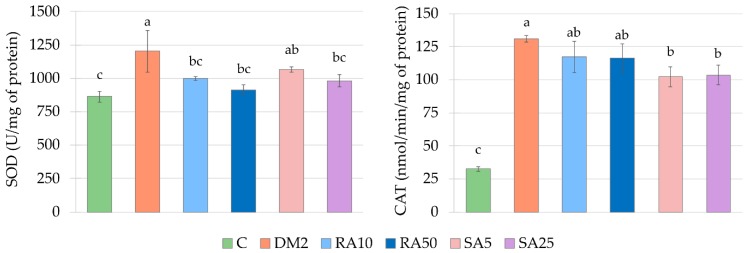
Effect of rosmarinic acid and sinapic acid on the antioxidative enzymes activities and non-enzymatic antioxidants level in the cardiac tissue of the HFD + STZ-induced type 2 diabetic rats. The results presented as mean ± SEM were evaluated by one-way ANOVA followed by LSD post-hoc test (statistically significant if *p* < 0.05). Values which have the same superscript letter(s) in each graph are not significantly different. If there are no letters in superscript, the ANOVA test was not significant. C: control rats; DM2: HFD + STZ-induced diabetic rats; RA10 and RA50: HFD + STZ-induced diabetic rats treated with rosmarinic acid at a dose of 10 mg/kg and 50 mg/kg, respectively; SA5 and SA25: HFD + STZ-induced diabetic rats treated with sinapic acid at a dose of 5 mg/kg and 25 mg/kg, respectively, for 28 days; HFD: high-fat diet; STZ: streptozotocin; SOD: superoxide dismutase; CAT: catalase.

**Figure 7:**
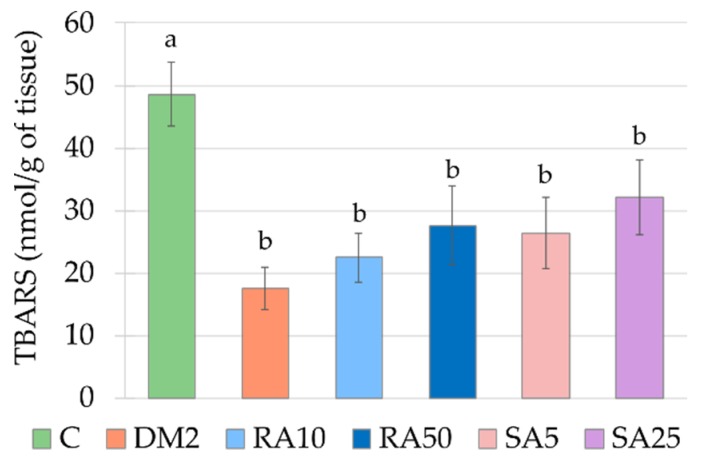
Effect of rosmarinic acid and sinapic acid on the oxidative damage markers level in the cardiac tissue of the HFD + STZ-induced type 2 diabetic rats. The results presented as mean ± SEM were evaluated by one−way ANOVA followed by LSD post-hoc test (statistically significant if *p* < 0.05). Values which have the same superscript letter(s) in each graph are not significantly different. When no letters are present in the superscript, the ANOVA test was not significant. C: control rats; DM2: HFD + STZ-induced diabetic rats; RA10 and RA50: HFD + STZ-induced diabetic rats treated with rosmarinic acid at a dose of 10 mg/kg and 50 mg/kg, respectively; SA5 and SA25: HFD + STZ-induced diabetic rats treated with sinapic acid at a dose of 5 mg/kg and 25 mg/kg, respectively, for 28 days; HFD: high-fat diet; STZ: streptozotocin; TBARS: thiobarbituric acid reactive substances.

The activities of SOD and CAT were significantly higher in the cardiac tissue of the DM2 rats than in the C rats, while the TBARS level was significantly lower. In comparison with the C rats, the GPx activity as well as the AOPP and NPSH levels remained unchanged in the cardiac tissue of the DM2 rats (Figure 6 and Figure 7, Appendix B, Table A3).

Both the doses of rosmarinic acid and the higher dose of sinapic acid decreased the activity of SOD in the cardiac tissue in comparison with the DM2 rats. The activity of SOD was not significantly different in the cardiac tissue of the rats from the RA10, RA50 and SA25 groups than in the C rats, but in the SA5 rats its activity was significantly higher than in the cardiac tissue of the C rats. There were no differences in the SOD activity between the groups of rats receiving phenolic acids. The CAT activity was significantly decreased in the cardiac tissue of the SA5 and SA25 rats in comparison with the DM2 rats, but this activity remained significantly higher than CAT activity in the cardiac tissue of the C rats. Despite the fact, that CAT activity was significantly lowered by administration of both the doses of sinapic acid when compared to the DM2 rats and was not lowered by any dose of rosmarinic acid, there were no significant differences in CAT activity in the cardiac tissue between the groups receiving rosmarinic acid and the groups treated with sinapic acid. The rest of the parameters connected with oxidative stress were not affected by administration of the tested phenolic acids (Figure 6 and Figure 7, Appendix B, Table A3).

### 3.8. PCA for the Cardiac Tissue

**Figure 8:**
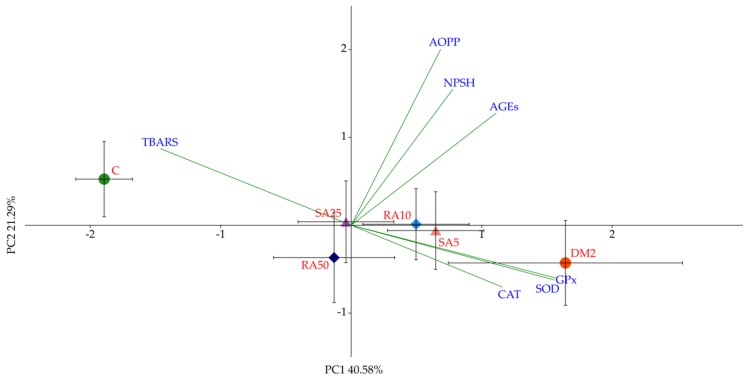
PCA biplot of parameters obtained from the heart tissue. Symbols and red fonts indicate means for individual experimental groups (whiskers represent SEM); ●—Control groups: green—untreated control rats (C); red—HFD + STZ control rats (DM2). ◆—Rosmarinic acid-treated HFD + STZ rats: light blue—10 mg/kg (RA10), navy blue – 50 mg/kg (RA50). ▲—Sinapic acid-treated HFD + STZ rats: pink—5 mg/kg (SA5), purple—25 mg/kg (SA25). Blue fonts and green lines show correlations of measured parameters with regard to experimental groups in the PCA plot (autoscaled).

Similarly to the results obtained from the serum, the PCA from the cardiac tissue showed a distinct separation of experimental groups across PC1, accounting for more than 40% of observed variation. The C and DM2 groups represented the two extremes, with C located all the way to the left, and DM2 all the way to the to the right of the aforementioned axis. The SA5 and RA10 groups clustered slightly to the right, closer to the DM2 group, whereas the SA25 and RA50 groups clustered near the center of the PC1 axis, with a slight shift towards the C group. However, it must be noted that although both clusters were located between the C and the DM2, only the separation of the latter one, RA50-SA25, can be considered significant with regard to both the C and DM2 (*p* < 0.001, LSD). The variation observed across PC1 was predominantly determined by the GPx, SOD, and CAT variables, correlating with the right portion of the plot, notably the DM2 group, as well as TBARS, which in turn correlated with the left portion of the plot and the C group. The separation across the PC2 was negligible as it did not produce any statistically significant differences between the groups (Figure 8).

**Figure 9:**
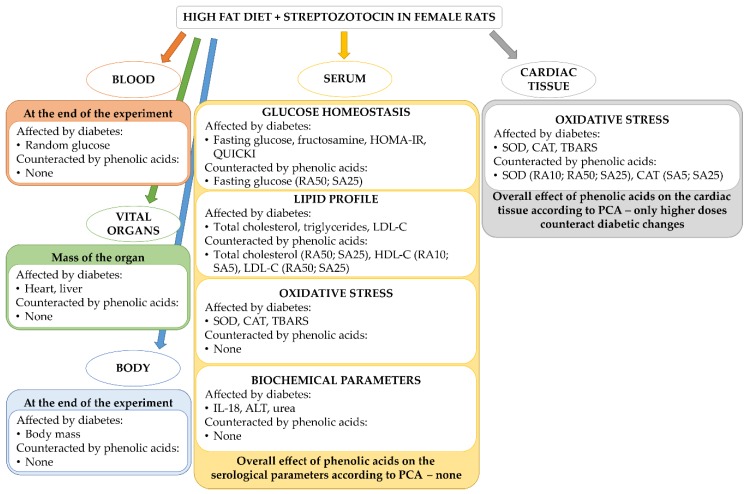
An overview of the parameters affected by HFD + STZ-induced type 2 diabetes and the effect of rosmarinic acid and sinapic acid on these changes. For each parameter counteracted by phenolic acid, the name and dose of the compound is given in the brackets: RA10 and RA50: rosmarinic acid administered at the doses of 10 and 50 mg/kg, respectively; SA5 and SA25: sinapic acid administered at the doses of 5 and 25 mg/kg, respectively. HOMA-IR: homeostasis model assessment of insulin resistance; QUICKI: quantitative insulin sensitivity check index; HDL-C: high-density lipoprotein cholesterol; LDL-C: low-density lipoprotein cholesterol; SOD: superoxide dismutase, CAT: catalase; TBARS: thiobarbituric acid reactive substances; IL-18: interleukin 18; ALT: alanine aminotransferase; PCA: principal component analysis.

Figure 9 outlines the main significant results obtained during the study—the effect of diabetes induced by HFD + STZ and the effect of the phenolic acids on the diabetes-induced changes. 

## 4. Discussion

In the presented study we aimed to evaluate the effect of rosmarinic acid and sinapic acid on oxidative stress-related parameters in the cardiac tissue as well as parameters connected with glucose homeostasis, lipid profile and oxidative stress in the serum of the rats with experimentally induced type 2 diabetes. Among the existing mice and rat models of diabetes, the model in which the animals are treated with a single injection of relatively high dose of streptozotocin is the most frequently used [50,51]. This model is characterized by a very high glucose level, polydipsia, polyuria, and, in consequence, dehydration and general exhaustion. The damage of the vital organs, which is a result of such a severe hyperglycemia, reflects type 1 diabetes rather than the type 2. There are several genetic models of the type 2 diabetes in rodents, such as: db/db mice or Zucker Diabetic Fatty (ZDF) rats (fa/fa), Goto Kakizaki (GK) rats, and Otsuka Long Evans Tokushima (OLET) rats. However, these genetic models are imperfect. This is due to the fact that insulin resistance, obesity and hyperglycemia in these models are the result of a gene mutation (e.g., leptin receptor OB-r), which can rarely be observed in humans [52]. Therefore, it can be concluded, that the model reflecting the changes observed in the type 2 diabetes in humans in the most adequate manner is a model in which a high-fat diet is combined with a single injection of a low dose of streptozotocin [52,53]. It should be noted that the majority of studies describing the effect of tested substances on parameters affected by both the type 1 and type 2 diabetes is based on the experiments conducted on male laboratory animals [17,19,20,21,22,23,24,25,32,33,34,35,37,38,50,51,54,55,56,57,58,59,60,61,62]. Following the suggestion of The National Institutes of Health (NIH) to use female animals in the laboratory studies [63] and reports about sex-dependent metabolic differences [64,65], our experiment was conducted on female rats. 

In our experiment, the diabetes was induced by a single injection of streptozotocin (40 mg/kg intraperitoneally) preceded by a 14-day treatment with a high-fat diet (32% of fat from lard). After streptozotocin injection, the high-fat diet was continued in the diabetic rats till the end of the experiment, i.e., for additional 35 days. This treatment induced disturbance in the glucose and lipid metabolism. Similarly to other reports, we observed increased random blood glucose level as well as the levels of fasting serum glucose, serum fructosamine, total cholesterol, and triglycerides. Moreover, induction of the type 2 diabetes resulted in the HOMA-IR increase and QUICKI decrease [32,35,54,56,57,58,66]. There were also changes in the oxidative stress-related parameters in the serum. Diabetes induced an increase in the SOD and CAT activities and in the level of the lipid peroxidation marker TBARS. The increase in the TBARS level is consistent with the other reports [58,59]. On the other hand, the reports on the effect of diabetes (including HFD + STZ-induced type 2 diabetes) on the activity of the antioxidant enzymes in the serum are incoherent. There are studies in which activity of these enzymes increases in the serum/plasma [21,55], and such which describe a decrease in the antioxidative enzymes activities [22,58,60]. Among other things, this discrepancy may be a result of differences in the duration to free radical exposure [67,68]. It should be noted that in our study neither AOPP (a marker for protein oxidative damage) nor NPSH (of which reduced glutathione (GSH), an endogenous nonenzymatic antioxidant, is the main representative) changed after induction of the type 2 diabetes. Numerous studies indicate that in the course of diabetes the GSH level in the serum/plasma decreases and the level of protein damage markers increases [21,22,32,57]. Lack of such alterations observed in our study may result from the fact that in females, even in the type 2 diabetes, estrogens play protective role against oxidative stress [69], and the estrogen level in rats in our study did not change significantly after induction of diabetes.

In this study the level of interleukin 18 (IL-18) was measured, since this interleukin is engaged in metabolic homeostasis and it is sex hormone-dependent [70,71]. The role of IL-18 in metabolic syndrome, obesity, or diabetes is not clear. On the one hand, in people with diabetes, metabolic syndrome, or obesity the IL-18 level is significantly higher than in healthy people [72]. On the other hand, in mice with inhibited IL-18 synthesis or with deficiency of inflammasome NLPR1, which is responsible for IL-18 synthesis, obesity, insulin resistance and other metabolic syndrome symptoms were observed [73,74]. We observed that in type 2 diabetic rats, which demonstrated insulin resistance, the level of IL-18 was significantly lower than in control healthy rats. Our results contradict the studies describing the elevated level of IL-18 in the Otsuka Long-Evans Tokushima Fatty rats and fructose-fed rats [75,76]. These differences may result from sexual dimorphism, as these studies were conducted on male rats [75,76] and not on female rats. Such gender discrepancies in the plasma IL-18 level were previously observed in wild-type mice injected intraperitoneally with LPS [77]. Another important factor which seems to affect the IL-18 level is diet. Russel et al. observed that the level IL-18 in the serum of ovariectomized rats depended on the isoflavone content in the chow [78]. However, our study is consistent with another report, in which the IL-18 level in Spontaneously Diabetic Torii rats was lower than in control, non-diabetic rats [79]. What is more, the authors of this study noticed, that IL-18 level differs in time after diabetes induction [79]. Taking into consideration all abovementioned literature data it could be assumed that IL-18 level may depend on many factors, including gender, species, strain or genetic model of the animal used for research, time point in which the measurement was taken, or even diet composition.

In the presented study, rosmarinic acid and sinapic acid were administered to the type 2 diabetic rats at the doses of 10 and 50 mg/kg and 5 and 25 mg/kg, respectively. The lower doses of the tested phenolic acids correspond with the dietary achievable doses. The 5-fold higher doses were chosen in order to evaluate if they may have a better therapeutic effect than the dietary doses. The same doses for both the phenolic acids were used previously in ovariectomized rats. Both rosmarinic acid and sinapic acid lowered HOMA-IR in ovariectomized rats and rosmarinic acid additionally lowered fructosamine level in these rats [39,40]. In the current study, in which rats were in a permanent hyperglycemic state, neither rosmarinic acid nor sinapic acid affected the parameters connected with glucose metabolism. Even though the higher doses of phenolic acids lowered the fasting glucose level in the serum, the fructosamine level in the serum (a parameter which reflects long-term glycemia status [80]), random blood glucose level or HOMA-IR were not decreased and QUICKI was not elevated. It should be noted that control of blood glucose level and monitoring the parameters representing long-term changes in glycemia (such as glycated hemoglobin A1c (HbA1c) or fructosamine) are a crucial element in prevention of diabetes complications [81,82,83]. It has been proven that postprandial glucose as well as random glucose levels have a closer association to glycated hemoglobin than the level of fasting plasma glucose [84,85,86]. As observed in a study conducted in type 2 diabetic patients, a greater emphasis should be put on regulation of the postprandial glucose level than on fasting glucose level, as it could produce better outcomes in general diabetes therapy e.g., lowering HbA1c [87]. 

Rosmarinic acid and sinapic acid administered to the type 2 diabetic rats at the higher doses (50 and 25 mg/kg respectively) lowered total cholesterol in the serum of the rats, similarly to rats in the early stage of estrogen deficiency [39,40]. Moreover, in our present study the higher doses of phenolic acids lowered the LDL-C in the serum of the type 2 diabetic rats. The beneficial effect of rosmarinic acid on lipid profile in rats with diabetes induced with high-fat diet and streptozotocin was previously shown [32,33]. In contrast to our results, in these works was shown that rosmarinic acid revealed a beneficial effect on parameters describing glucose metabolism and oxidative stress [32,33,35]. In two of these studies, administration of rosmarinic acid at a dose of 100 mg/kg resulted in a decrease in glycated hemoglobin and HOMA-IR [32,35] and in the third study, rosmarinic acid administered at a dose of 30 mg/kg lowered the AGEs level in the heart, liver, and kidney [33]. There is also a study in which sinapic acid administered at the dose of 25 mg/kg to the HFD + STZ-induced diabetic rats demonstrated a positive effect on glucose metabolism parameters such as: HbA1c or HOMA-IR [37]. However, it should be highlighted that all the aforementioned studies [32,33,35,37] were conducted on male rats and not on female rats like in our study. Therefore, it is possible that inconsistency in rosmarinic acid action results from sex differences. To the best of our knowledge, there were no studies describing the effect of sinapic acid on parameters connected with lipid profile in diabetic animals. Positive effect of sinapic acid on lipids level in the serum/plasma was observed in different experimental models, such as isoproterenol-induced myocardial infarcted rats [44] or L-NAME-induced hypertensive rats [43].

Based on the PCA it was determined that the variables predominantly responsible for the variance observed between the C group and all of the diabetic groups of rats were fasting glucose, fructosamine, HOMA-IR, QUICKI, and IL-18. There were no differences in these variables after the phenolic acids administration, thus no separation between these groups (RA10, RA50, SA5, and SA25) or DM2 was observed in the PCA. Even though there were minor differences in individual lipid profile markers, they were too weak to demonstrate the visible effect on the PCA, as much stronger effects were related to glucose metabolism. It is worth noting that random blood glucose measured in the capillary vessels was not affected after the phenolic acid administration for 28 days. This is in agreement with the PCA results. 

The results for oxidative stress parameters obtained from the cardiac tissue of the HFD + STZ-induced type 2 diabetic rats are surprising. The TBARS level in this tissue was significantly lower than in the control rats. The majority of the authors reports that the lipid peroxidation markers level increases in the heart during diabetes induced with streptozotocin alone [26,51] or with high-fat diet combined with streptozotocin [27,33,88]. Only few publications indicate that TBARS level does not change or decreases in the cardiac tissue in diabetic rodents [61,89]. The lowered TBARS level in the heart was observed by the authors, who fed the rats with high-fat diet (with 30% of fat from palm oil) for 11 weeks [90]. These authors explain the result by changes in polyunsaturated fatty acids (PUFA) profile in phospholipids of the heart membrane—there was a significant increase in arachidonic acid (AA) in the heart membrane of rats on palm oil-rich diet, compared to the membrane of rats fed with standard chow [90]. In our study, rats were fed with diet enriched with 32% of fat from lard. The same research team who examined palm oil-rich diet in another study tested the changes in PUFA profiles after lard-rich diet. Similarly to palm oil-rich diet, the diet rich in lard also effected in an increase of AA in the heart membrane [91]. The authors suggest that AA has a strong effect on vessel dilatation and its accumulation may promote coronary flow, which can result in an increased mechanical function of the heart [90].

In the cardiac tissue, similarly to the serum, the activity of antioxidative enzymes was elevated. These results are in line with some other reports [26,61], but there are also reports indicating a decrease in antioxidative enzymes activity in the heart of diabetic animals [24,33,58,62]. In the study conducted by Okutan et al. regarding the effect of caffeic acid phenethyl ester on oxidative stress markers in the heart it was shown that similarly to our results, the activities of SOD and CAT were elevated in diabetic rats, while GPx activity was unchanged when compared to the non-diabetic animals. After administration of caffeic acid phenethyl ester, the activities of SOD and CAT was lowered. The authors concluded that administration of this derivative of phenolic acid reduced the diabetes-induced oxidative stress in the heart [26]. Therefore, based on these findings, we also may conclude that lowering of SOD activity by both the doses of rosmarinic acid and the higher dose of sinapic acid, as well as lowering of CAT activity by both the doses of sinapic acid indicate a positive effect of these phenolic acids on cardiac tissue of type 2 diabetic rats.

In our experiment we have observed a diverse effect of individual phenolic acids on oxidative stress parameters in the cardiac tissue. Furthermore, for each compound there were discrepancies in the effects of individual doses on specific markers of oxidative stress. However, the multivariate analysis performed on the cardiac tissue-derived variables has confirmed a negligible or no effect of phenolic acids when administered in dietary doses of 10 mg/kg and 5 mg/kg for rosmarinic and sinapic acid, respectively. However, it should be noted that higher doses of the aforementioned compounds did beneficially affect the oxidative stress markers, as evidenced by the PCA. These differences were masked in individual ANOVAs. This might be due to the fact that classic analysis of variance takes into consideration only quantitative characteristics of measured variables, whereas the specifics of the PCA introduce a qualitative component to the study, as the effect (weight) of individual variables on the total observed variation between experimental groups along PC1 and PC2 axes is not equal [92].

In the current research we focused on biochemical parameters in the serum and oxidative stress markers in the serum and cardiac tissue in the female diabetic rats. Due to this fact, this study has several limitations. Firstly, we did not conduct histological analyses or examine the molecular mechanisms underlying the action of tested phenolic acids in the cardiac tissue. We did not evaluate the effect of diabetes and phenolic acids administration on the markers connected to inflammation (e.g., IL-1β, IL-6, IL-10, IL-18, tumor necrosis factor α), apoptosis (for instance caspase 3 or p53), or cellular function (such as adenosine monophosphate-activated protein kinase, mitochondrial transcription factor A, or phosphoinositide 3-kinase) in the cardiac tissue. Secondly, further studies regarding rosmarinic acid or sinapic acid should include the cardiac mechanical function. Finally, in order to find out whether sex hormone environment is an important factor in the response to phenolic acids treatment, the next experiment should be conducted on both male and female rats. 

## 5. Conclusions

Based on the presented study, it can be concluded that rosmarinic and sinapic acids did not affect glucose homeostasis or oxidative stress parameters in the serum. As a whole, the effects visible in the serum upon phenolic acid administration to the type 2 diabetic female rats can be considered minor. Only few specific parameters related to the lipid profile were slightly improved after administration of these plant-derived compounds. 

The effect of rosmarinic and sinapic acids on oxidative stress parameters in the cardiac tissue was demonstrated only when higher doses were administered, whereas the effect of dietary-achievable doses of these compounds on tested parameters was negligible in the type 2 diabetic female rats. Therefore, we suggest conducting further studies considering rosmarinic and sinapic acids focused on doses higher than achievable during the regular nutrition regimen, which might have a therapeutic effect in the cardiac tissue of diabetic subjects.

Our study shows that multivariate analyses such as PCA can be considered useful for capturing relationships between all measured variables and the effects of pharmacologically active substances in model organisms and in various experimental set-ups.

## Figures and Tables

**Table 1:** Effect of rosmarinic acid and sinapic acid on the glucose homeostasis-related parameters in the serum and cardiac tissue in HFD + STZ-induced type 2 diabetic rats.

Parameter	C	DM2	RA10	RA50	SA5	SA25
Serum fasting glucose (mg/dL)	98.4 ± 4.8 ^c^	309.7 ± 18.5 ^a^	316.9 ± 18.0 ^a^	239.1 ± 16.7 ^b^	303.3±20.8 ^a^	253.8 ± 15.1 ^b^
Serum fasting insulin (µU/mL)	13.99 ± 2.06	11.89 ± 1.47	12.95 ± 2.21	13.42 ± 0.98	13.25±1.40	13.75 ± 1.60
HOMA-IR	3.14 ± 0.52 ^b^	9.20 ± 1.41 ^a^	9.25 ± 1.32 ^a^	7.86 ± 0.79 ^a,b^	9.94±1.56 ^a^	8.77 ± 1.43 ^a^
QUICKI	0.326 ± 0.007 ^a^	0.283 ± 0.005 ^b^	0.282 ± 0.005 ^b^	0.286 ± 0.003 ^b^	0.280±0.006 ^b^	0.285 ± 0.005 ^b^
Serum fructosamine (mmol/L)	0.563 ± 0.019 ^b^	0.639 ± 0.023 ^a^	0.642 ± 0.021 ^a^	0.659 ± 0.019 ^a^	0.665 ± 0.011 ^a^	0.683 ± 0.025 ^a^
Serum AGEs (µg/mL)	33.28 ± 1.05	37.00 ± 0.91	35.72 ± 1.80	36.83 ± 2.02	35.54 ± 2.97	37.56 ± 1.29
Cardiac AGEs (µg/g)	25.93 ± 1.36	33.84 ± 2.91	29.18 ± 3.27	28.93 ± 3.03	31.40 ± 3.03	27.94 ± 2.10

The results presented as mean ± SEM were evaluated by one-way ANOVA followed by LSD post-hoc test (statistically significant if *p* < 0.05). Values which have the same superscript letter(s) within each row are not significantly different. When no letters are present in the superscript, the ANOVA test was not significant. C: control rats; DM2: HFD + STZ-induced diabetic rats; RA10 and RA50: HFD + STZ-induced diabetic rats treated with rosmarinic acid at a dose of 10 mg/kg and 50 mg/kg, respectively; SA5 and SA25: HFD + STZ-induced diabetic rats treated with sinapic acid at a dose of 5 mg/kg and 25 mg/kg, respectively, for 28 days; HOMA-IR: homeostasis model assessment of insulin resistance; QUICKI: quantitative insulin sensitivity check index; AGEs: advanced glycation end products.

**Table 2:** Effect of rosmarinic acid and sinapic acid on the oxidative stress-related parameters in the serum of HFD + STZ-induced type 2 diabetic rats.

Parameter	C	DM2	RA10	RA50	SA5	SA25
SOD (U/mg of protein)	7.06 ± 0.67 ^b^	9.08 ± 0.58 ^a^	8.56 ± 0.62 ^a,b^	10.29 ± 0.32 ^a^	9.92 ± 0.56 ^a^	9.76 ± 0.60 ^a^
CAT (nmol/min/mg of protein)	0.389 ± 0.074 ^b^	0.943 ± 0.142 ^a^	0.690 ± 0.084 ^a,b^	1.084 ± 0.292 ^a^	0.907 ± 0.129 ^a^	0.933 ± 0.131 ^a^
NPSH (nmol/mL)	1.90 ± 0.18	1.22±0.08	2.03 ± 0.26	1.63 ± 0.15	1.86 ± 0.26	1.72 ± 0.16
TBARS (nmol/mL)	11.91 ± 2.01 ^b^	24.95 ± 4.34 ^a^	23.27 ± 4.07 ^a^	23.19 ± 3.17 ^a^	20.76 ± 2.11 ^a^	18.53 ± 2.08 ^a,b^
AOPP (nmol/mL)	20.94 ± 2.73	65.60 ± 18.67	74.05 ± 17.54	71.31 ± 28.99	54.13 ± 18.11	62.26 ± 35.62

The results presented as mean ± SEM were evaluated by one-way ANOVA followed by LSD post-hoc test (statistically significant if *p* < 0.05). Values which have the same superscript letter(s) within each row are not significantly different. When no letters are present in the superscript, the ANOVA test was not significant. C: control rats; DM2: HFD + STZ-induced diabetic rats; RA10 and RA50: HFD + STZ-induced diabetic rats treated with rosmarinic acid at a dose of 10 mg/kg and 50 mg/kg, respectively; SA5 and SA25: HFD + STZ-induced diabetic rats treated with sinapic acid at a dose of 5 mg/kg and 25 mg/kg, respectively, for 28 days; SOD: superoxide dismutase; CAT: catalase; NPSH: non-protein sulfhydryl groups; TBARS: thiobarbituric acid reactive substances; AOPP: advanced oxidation protein products.

## Appendix A

### Appendix A.1. SERUM

MANOVA–PCA score coordinates (axes combined).

Effect	Test	Value	F	Effect df	Error df	*p*
Group	Wilks	0.19497	10.11805	10	80	0.00000

PCA axes individually.

Effect	df	PC1				PC2			
		SS	MS	F	*p*	SS	MS	F	*p*
Group	5	185.7365	37.1473	25.9662	0.0000	22.7688	4.5538	1.6953	0.1574
Error	41	58.6548	1.4306			110.1278	2.6860		
Total	46	244.3913				132.8967			

LSD post-hoc axis 1.

Group	PC1 Mean	a	b
C	−3.79521		****
SA25	0.65955	****	
RA50	0.71558	****	
RA10	1.07581	****	
SA5	1.15881	****	
DM2	1.39560	****	

LSD post-hoc axis 2—not significant.

### Appendix A.2. CARDIAC TISSUE

MANOVA–PCA score coordinates (axes combined).

Effect	Test	Value	F	Effect df	Error df	*p*
Group	Wilks	0.456318	3.650709	10	76	0.00052

PCA axes individually.

Effect	df	PC1				PC2			
		SS	MS	F	*p*	SS	MS	F	*p*
Group	5	59.7301	11.9460	7.1402	0.0001	4.8962	0.9792	0.6294	0.1574
Error	39	65.2491	1.6731			60.6784	1.5559		
Total	44	124.9792				65.5746			

LSD post-hoc axis 1.

Group	PC1 Mean	b	a	c
C	−1.89318			****
RA50	−0.13118	****		
SA25	−0.04002	****		
RA10	0.49691	****	****	
SA5	0.64746	****	****	
DM2	1.64162		****	

LSD post-hoc axis 2—not significant.

## Appendix B

**Table A1:** Effect of rosmarinic acid and sinapic acid on the vital organ mass in HFD + STZ-induced type 2 diabetic rats.

Parameter	C	DM2	RA10	RA50	SA5	SA25
Heart mass (g)	0.607 ± 0.014 ^c^	0.662 ± 0.017 ^a,b^	0.639 ± 0.013 ^b,c^	0.628 ± 0.009^bc^	0.645 ± 0.016 ^a,b,c^	0.694 ± 0.030 ^a^
Liver mass (g)	5.972 ± 0.204 ^b^	7.178 ± 0.245 ^a^	7.107 ± 0.181 ^a^	6.695 ± 0.247^a^	7.069 ± 0.197 ^a^	6.810 ± 0.139 ^a^
Kidney mass (g)	0.764 ± 0.022 ^c^	0.822 ± 0.032 ^a,bc^	0.854 ± 0.015 ^a,b^	0.823 ± 0.039 ^a,b,c^	0.886 ± 0.028 ^a^	0.802 ± 0.012 ^b,c^
Uterus mass (g)	0.425 ± 0.039	0.290 ± 0.038	0.339 ± 0.045	0.455 ± 0.068	0.419 ± 0.075	0.459 ± 0.039

The results presented as mean ± SEM were evaluated by one-way ANOVA followed by LSD post-hoc test (statistically significant if *p* < 0.05). Values which have the same superscript letter(s) within each row are not significantly different. When no letters are present in the superscript, the ANOVA test was not significant. C: control rats; DM2: HFD + STZ-induced diabetic rats; RA10 and RA50: HFD + STZ-induced diabetic rats treated with rosmarinic acid at a dose of 10 mg/kg and 50 mg/kg, respectively; SA5 and SA25: HFD + STZ-induced diabetic rats treated with sinapic acid at a dose of 5 mg/kg and 25 mg/kg, respectively, for 28 days.

**Table A2:** Effect of rosmarinic acid and sinapic acid on the biochemical parameters in the serum of HFD + STZ-induced type 2 diabetic rats.

Parameter	C	DM2	RA10	RA50	SA5	SA25
Estradiol (pg/mL)	43.84 ± 10.84	22.41 ± 2.03	18.64 ± 1.92	31.49 ± 5.07	21.67 ± 5.34	34.58±8.29
Interleukin-18 (pg/mL)	348.8 ± 21.8 ^a^	141.9 ± 8.8 ^b^	190.2 ± 26.5 ^b^	149.4 ± 15.1 ^b^	198.0 ± 30.2 ^b^	172.1 ± 7.1 ^b^
AST (U/L)	36.54 ± 3.54	33.25 ± 4.92	31.27 ± 2.91	36.70 ± 5.80	38.11 ± 3.81	37.36 ± 3.52
ALT (U/L)	5.53 ± 0.64 ^c^	11.81 ± 1.26 ^a,b^	11.97 ± 2.23 ^a,b^	10.05 ± 1.14 ^b^	16.61 ± 2.80 ^a^	13.32 ± 1.54 ^a,b^
Uric acid (mg/dL)	1.05 ± 0.06	2.11 ± 0.40	2.74 ± 0.59	2.27 ± 0.89	2.88 ± 0.73	1.29 ± 0.19
Urea (mg/dL)	38.87 ± 2.02 ^a^	29.79 ± 1.83 ^b^	29.87 ± 3.19 ^b^	32.60 ± 3.55 ^a,b^	33.50 ± 3.53 ^a,b^	26.58 ± 2.29 ^b^
Creatinine (mg/dL)	0.466 ± 0.017	0.445 ± 0.010	0.469 ± 0.038	0.446 ± 0.018	0.413 ± 0.012	0.441 ± 0.006

The results presented as mean ± SEM were evaluated by one-way ANOVA followed by LSD post-hoc test (statistically significant if *p* < 0.05). Values which have the same superscript letter(s) within each row are not significantly different. When no letters are present in the superscript, the ANOVA test was not significant. C: control rats; DM2: HFD + STZ-induced diabetic rats; RA10 and RA50: HFD + STZ-induced diabetic rats treated with rosmarinic acid at a dose of 10 mg/kg and 50 mg/kg, respectively; SA5 and SA25: HFD + STZ-induced diabetic rats treated with sinapic acid at a dose of 5 mg/kg and 25 mg/kg, respectively, for 28 days; AST: aspartate aminotransferase; ALT: alanine aminotransferase.

**Table A3:** Effect of rosmarinic acid and sinapic acid on the oxidative stress-related parameters in the cardiac tissue of HFD + STZ-induced type 2 diabetic rats.

Parameter	C	DM2	RA10	RA50	SA5	SA25
GPx (nmol/min/mg of protein)	0.900 ± 0.055	1.298 ± 0.208	1.066 ± 0.051	1.048 ± 0.047	1.175 ± 0.047	1.070 ± 0.075
NPSH (nmol/g of tissue)	19.50 ± 2.09	19.08 ± 2.53	29.53 ± 5.12	20.04 ± 5.09	29.14 ± 3.52	25.61 ± 4.83
AOPP (nmol/mg of protein)	27.87 ± 3.26	31.44 ± 5.94	27.77 ± 3.02	25.96 ± 3.25	24.87 ± 2.70	28.10 ± 2.10

The results presented as mean ± SEM were evaluated by one-way ANOVA followed by LSD post-hoc test (statistically significant if *p* < 0.05). Values which have the same superscript letter(s) within each row are not significantly different. When no letters are present in the superscript, the ANOVA test was not significant. C: control rats; DM2: HFD + STZ-induced diabetic rats; RA10 and RA50: HFD + STZ-induced diabetic rats treated with rosmarinic acid at a dose of 10 mg/kg and 50 mg/kg, respectively; SA5 and SA25: HFD + STZ-induced diabetic rats treated with sinapic acid at a dose of 5 mg/kg and 25 mg/kg, respectively, for 28 days; GPx: glutathione peroxidase; NPSH: non-protein sulfhydryl groups; AOPP: advanced oxidation protein products.
